# Prevalence of cannabis use for pain management in Quebec: A post-legalization estimate among generations living with chronic pain

**DOI:** 10.1080/24740527.2022.2051112

**Published:** 2022-06-03

**Authors:** Marimée Godbout-Parent, Hermine Lore Nguena Nguefack, Adriana Angarita-Fonseca, Claudie Audet, Andréanne Bernier, Ghita Zahlan, Nancy Julien, M. Gabrielle Pagé, Line Guénette, Lucie Blais, Anaïs Lacasse

**Affiliations:** a Département des sciences de la santé, Université du Québec en Abitibi-Témiscamingue (UQAT), Rouyn-Noranda, Québec, Canada; b Centre de recherche du Centre hospitalier de l’Université de Montréal (CRCHUM), Montréal, Québec, Canada; c médecine, Université de MontréalDépartement d’anesthésiologie et de médecine de la douleur, Faculté de, Montréal, Québec, Canada; d Faculté de pharmacie, Université Laval, Québec, Québec, Canada; e Centre de recherche du CHU de Québec–Université Laval, Québec, Québec, Canada; f Faculté de pharmacie, Université de Montréal, Montréal, Québec, Canada

**Keywords:** cannabis, cannabinoids, chronic pain, prevalence, age, generations, associated factors, determinants, gender

## Abstract

**Background:**

Medical cannabis has been legal in Canada since 2001, and recreational cannabis was legalized in October 2018, which has led to a widespread increase in the accessibility of cannabis products.

**Aims:**

This study aimed to estimate the prevalence of cannabis use among adults living with chronic pain (CP) and investigate the relationship between age and cannabis use for CP management.

**Methods:**

A cross-sectional analysis of the COPE Cohort data set, a large Quebec sample of 1935 adults living with CP, was conducted. Participants completed a web-based questionnaire in 2019 that contained three yes/no questions about past-year use of cannabis (i.e., for pain management, management of other health-related conditions, recreational purposes).

**Results:**

Among the 1344 participants who completed the cannabis use section of the questionnaire, the overall prevalence of cannabis use for pain management was 30.1% (95% confidence interval 27.7–32.7). Differences were found between age groups, with the highest prevalence among participants aged ≤26 years (36.5%) and lowest for those aged ≥74 years (8.8%). A multivariable logistic model revealed that age, region of residence, generalized pain, use of medications or nonpharmacological approaches for pain management, alcohol/drug consumption, and smoking were associated with the likelihood of using cannabis for pain management.

**Conclusions:**

Cannabis is a common treatment for the management of CP, especially in younger generations. The high prevalence of use emphasizes the importance of better knowledge translation for people living with CP, rapidly generating evidence regarding the safety and efficacy of cannabis, and clinicians’ involvement in supporting people who use cannabis for pain management.

## RÉSUMÉ

**Contexte:** Le cannabis médical est légal au Canada depuis 2001 et le cannabis récréatif a été légalisé en octobre 2018, ce qui a conduit à une augmentation généralisée de l'accessibilité des produits du cannabis. **Objectifs:** Cette étude visait à estimer la prévalence de la consommation de cannabis chez les adultes vivant avec la douleur chronique et à étudier l'association entre l'âge et la consommation de cannabis pour la prise en charge de la douleur chronique. **Méthodes:** Une analyse transversale de l'ensemble de données de la cohorte COPE, un grand échantillon québécois de 1 935 adultes vivant avec la douleur chronique, a été menée. En 2019, les participants ont rempli un questionnaire en ligne qui contenait trois questions oui/non sur la consommation de cannabis au cours de l'année écoulée (c.-à-d., pour la prise en charge de la douleur, la prise en charge d'autres affections liées à la santé, à des fins récréatives). **Résultats:** Parmi les 1 344 participants qui ont rempli la section du questionnaire portant sur la consommation de cannabis, la prévalence globale de la consommation de cannabis pour la prise en charge de la douleur était de 30,1 % (intervalle de confiance à 95 %, 27,7-32,7). Des différences ont été constatées entre les groupes d'âge, avec la prévalence la plus élevée chez les participants âgés de ≤ 26 ans (36,5 %) et la plus basse chez les participants âgés de ≥ 74 ans (8,8 %). Un modéle logistique multivariable a révélé que l'âge, la région de résidence, la douleur généralisée, l'utilisation de médicaments ou approches non pharmacologiques pour la prise en charge de la douleur, la consommation d'alcool/de drogue et le tabagisme étaient associés à la probabilité d'utiliser le cannabis pour la prise en charge de la douleur. **Conclusions:** Le cannabis est un traitement courant pour la prise en charge de la douleur chronique, en particulier chez les jeunes générations. La prévalence élevée de l'utilisation souligne l'importance d'un meilleur transfert des connaissances pour les personnes vivant avec la douleur chronique, en générant rapidement des donnant probantes concernant l'innocuité et l'efficacité du cannabis, ainsi que l'implication des cliniciens dans le soutien aux personnes qui consomment du cannabis pour la prise en charge de la douleur.

## Introduction

Despite limited evidence of the safety and efficacy of cannabis as a treatment, chronic pain (CP) is among the most frequent health-related conditions for which cannabis is used.^1–3^ Medical cannabis has been legal in Canada since 2001, and recreational use was legalized toward the end of October 2018.^4^ Following this new legislation, social acceptability has increased^5^ and consumers have experienced improved access to both recreational and medical cannabis.^6^

Evidence regarding the benefits of using cannabis for CP management is limited and contradictory. Though some studies suggested advantages, a recent meta-analysis reported small to very small improvements in terms of pain, functioning, and sleep.^7^ Physical and mental harms are also present, such as dizziness, nausea, vomiting, drowsiness, impaired attention, and transient cognitive impairment.^7^ Due to significant gaps in research regarding the safety and efficacy of cannabis and cannabinoids for the management of pain, the International Association for the Study of Pain Presidential Task Force^8,9^ does not currently support its use. Their work highlights preliminary evidence for cannabinoid-induced analgesia in preclinical investigations but emphasizes that no evidence of sufficient quality currently exists in clinical populations.^8^ This echoes the position statement from the Canadian Rheumatology Association^10^ and UK National Institute for Clinical Excellence.^11^ A number of factors complicate the review of the current literature or conducting new investigations on the use of cannabis and cannabinoids for the management of CP, including the great variability of types of cannabinoids used, route of administration, dosage, types of populations, types of pain, as well as the quality of the research currently published.^12^ Despite published position statements and concerns, the attitudes of patients with CP toward cannabis products appear to be highly favorable.^13,14^ An international panel including representatives from relevant medical and nonmedical specialties in addition to methodologists and patients recently recommend that if standard care and other treatment options are not sufficient, a trial of noninhaled medical cannabis or cannabinoids could be suggested to patients (with the knowledge that the improvements in pain and sleep will probably be small). They were in favor of cannabis use for CP management with some careful follow-up (e.g., start with low doses, carefully monitor adverse events, avoid driving, etc.).^15^

The pre-legalization prevalence of cannabis use in Canadians living with CP was estimated to be 10% or less (studies conducted before October 2018).^16,17^ In Ware and colleagues’ study, 10% of participants reported current use of cannabis for pain relief, and results further highlighted a wide range of frequency of use, dosage, mode of administration, and type of cannabis product.^16^ A few years prior to the legalization of recreational cannabis, Ste-Marie and colleagues’ study of individuals living with rheumatic diseases reported that 4.3% of participants had ever used medical cannabis, with 2.8% reporting continuing use.^17^ They repeated the same study method post-legalization and found a prevalence of 12.6% of individuals who had ever used medical cannabis, with 6.5% reporting current medical use.^18^ Their results suggested a twofold increase in prevalence of medical use of cannabis following the legalization of recreational cannabis. However, it is relevant to expand investigations about the current prevalence of cannabis use in large and diversified samples of Canadians living with CP in this post-legalization environment.

In order to have a more complete picture, particular attention should also be given to sociodemographic factors that may predict cannabis use among people living with CP. According to some studies conducted in the general population and published before the legalization of recreational use, age appeared to be a factor influencing the use of cannabis, alongside other sociodemographic factors, such as sex, tobacco consumption, race/ethnicity, marital status, and other socioeconomic factors.^19^ Though some Canadian general population studies showed no substantial variation across age groups,^20^ a 2015 report observed a lower prevalence of cannabis use among older people.^1^ It should be noted that limited post-legalization data specific to Canadian CP populations is available regarding the influence of age and other sociodemographic factors on cannabis use.

The goals of the present study were to (1) measure the post-legalization prevalence of cannabis use among Canadians living with CP and explore the reasons for use, (2) compare the prevalence of cannabis use for CP management among generations/age groups, and (3) investigate the relationship between age and cannabis use for CP management adjusted by several sociodemographic and clinical factors.

## Methods

### Data Source

The present study utilized data from the ChrOnic Pain trEatment (COPE) Cohort,^21^ which is a data set designed to further our understanding of real-world usage of pharmacological as well as of physical and psychological treatments among people living with CP. The COPE Cohort includes 1935 French-speaking adults from the province of Quebec (Canada) who self-reported living with CP (persistent or recurrent pain for more than three months^22^). Between June and October 2019, participants completed a web-based questionnaire that included previously used items and validated composite scales. All indicators identified as a minimum data set by the Canadian Registry Working Group of the Strategy for Patient-Oriented Research Chronic Pain Network^23^ were included: pain location, circumstances surrounding its onset, duration, frequency, intensity, neuropathic component, interference, physical function, anxiety and depressive symptoms, age, sex, gender, and employment status. Item selection was also guided by the core outcome domains and measures by the Initiative on Methods, Measurement and Pain Assessment in Clinical Trials,^24,25^ items of the Canadian minimum data set for chronic low back pain research,^26^ and variables assessed in the Quebec Pain Registry.^27^ Self-reported data were also intended to be linked to longitudinal administrative data (medical and prescription claims). The complete methodology of the COPE cohort implementation is described elsewhere.^21^

In terms of representativeness, online self-reported data collection enabled the research team to reach many participants from all regions of the province of Quebec (including several remote understudied regions), reduce social desirability bias,^28^ as well as minimize data entry errors. COPE cohort participants’ pain characteristics, age, employment status, and level of education have been found to be representative of other large random samples of adults living with CP in Canada and elsewhere.^29–34^ However, the COPE cohort was found to overrepresent individuals having access to the Internet, women, and users of pain medications.^21^ The online recruitment strategy and questionnaire administration could explain the oversampling of women, because they use Facebook^35^ and work in online environments more often than men.^36^ Women also use more prescribed medications.^37^ Nevertheless, the COPE cohort still includes a diverse spectrum of profiles, allowing the assessment of valid multivariable associations. For prevalence estimates, calculating gender-stratified measures reduces the possibility of bias that may arise from the overrepresentation of women.

### Informed Consent Statement

The present study received ethics approval from the Université du Québec en Abitibi-Témiscamingue’s research ethics committee (#2018-05, Lacasse, A.). Participants completed informed consent for participation online and were informed that they would not be identifiable from the published results.

### Selection Criteria

This study was conducted using self-reported data among the sample of participants who completed the cannabis use section of the questionnaire (*n* = 1344/1935). Participants included in the study were clinically comparable to those not included (*n* = 591/1935) in terms of mean age (49.72 versus 52.3 years old), proportion of women (83.5% versus 86.3%), and proportion of individuals reporting moderate to severe pain in the past 7 days (67.7% versus 70.9%).

### Study Variables

*Past-year use of cannabis*. The cannabis use section of the questionnaire was made up of three yes/no questions about past-year use of cannabis (i.e., for pain management, for the management of other health-related conditions, for recreational purposes; non-mutually exclusive prevalence estimates). Overall prevalence of use was computed by calculating the percentage of participants who answered “yes” to at least one of these three questions.

*Age groups*. Age groups were formed based on recognized generational groups in Canada^38^ applied to the year of study recruitment (2019): (1) Generation Z (≤26 years old), (2) children of baby boomers (27–47 years old, which include millennials/Generation Y), (3) baby busters (48–53 years old, which includes Generation X), (4) baby boomers (54–73 years old), and (5) parents of baby boomers (≥74 years old, also known as traditionalists or the silent generation). This categorization was chosen because we felt that it would reflect different social norms that might influence cannabis use.

*Covariates*. We considered the following covariates: sociodemographic characteristics (gender identity, gender roles, country of birth, Indigenous identity, employment status, disability, education level, region of residence), CP characteristics (generalized pain, multisite pain, pain frequency, duration, intensity, tendency toward pain catastrophizing, neuropathic component, pain interference), pain management (current use of prescribed pain medications, over-the-counter/nonprescribed pain medications, or nonpharmacological treatments; access to a trusted health care professional for pain management), and health profile and lifestyle variables (psychological distress, perceived general health, number of medications currently used, physical functioning, alcohol or drug use, cigarette smoking). All COPE self-reported measurements and validated composite scales are described elsewhere.^21^

### Statistical Analysis

The characteristics of the study population and cannabis use (overall, for pain management, for other health-related conditions, for recreational purposes) were summarized using descriptive statistics. Prevalence of cannabis use (overall and for pain management) was then compared across the abovementioned age groups. As per best sex- and gender-based analysis practices guidelines,^39^ prevalence estimates were also stratified across gender identity subgroups (women, men, nonbinary). Chi-square and Fisher’s exact tests, in addition to post hoc multiple comparisons (Tukey-style multiple comparisons of proportions), were conducted.

A multivariable logistic regression model was used to investigate the relationship between age and cannabis use for CP management. The COPE cohort variables that could potentially be related to age and/or cannabis use were identified a priori and were included in the regression analysis. These variables were chosen based on existing literature and clinical considerations. Our substantial sample size allowed us to favor this approach versus other criticized covariate selection techniques (e.g., relying on bivariate regression analyses, *P* values,^40^ or computer algorithms^41^). Variance inflation factors were below 2.5 for all variables included in the multivariable model (variance inflation factors <5 or 10 are often suggested to detect multicollinearity^42^). The Hosmer-Lemeshow test confirmed a well-fitting model (chi-square = 7.2; *P* = 0.5190). Regression results are presented as adjusted odds ratios (ORs) along with their 95% confidence intervals (CIs). Because interpreting ORs quantitatively can be misleading when the underlying outcome is common and when effect sizes are modest to strong,^43^ they were interpreted qualitatively (i.e., presence of a statistically significant association and its direction rather than its magnitude). A sensitivity analysis was also carried out to assess the impact of missing data imputation on conclusions. Multiple regression analyses were used to estimate missing values^41^ (five different imputations) and our model was run again using those data sets as a sensitivity analysis. Analyses were conducted using SAS version 9.4 (SAS Institute, Cary, NC, USA).

## Results

Table 1 presents the characteristics of the sample. Among the 1344 participants who were included in the present study, 83.5% self-identified as women, 16.2% as men and 0.3% as nonbinary (*n* = 4). The average age 49.7 (SD ±13.2 years, range 18–88 years) and our sample was included mostly children of baby boomers (27–47 years, which include millennials/Generation Y; 40.4%) and baby boomers (54–73 years; 38.1%). Most had a postsecondary education (79.7%) and were not employed (63.3%). Most of the sample reported multisite pain (88.7%), with 74.3% reporting living with pain for at least 5 years. A majority reported moderate to severe pain (67.7%) and reported taking prescribed pain medications (79.9%) and/or over-the-counter pain medications (67.4%) for the management of their pain.

**Table 1.** Characteristics of the sample.

Characteristics (*n* = 1344)	*n* (%)^a^
Age (years), mean ± standard deviation Range	49.72 ± 13.17 18–88
Generation/age group	
Generation Z (≤26 years)	52 (3.92)
Children of baby boomers (27–47 years)	535 (40.38)
Baby busters (48–53 years)	199 (15.02)
Baby boomers (54–73 years)	505 (38.11)
Parents of baby boomers (≥74 years)	34 (2.57)
Self-identified gender Woman Man Nonbinary	1122 (83.54) 217 (16.16) 4 (0.29)
Employed Yes No	491 (36.70) 847 (63.30)
Postsecondary education Yes No	1064 (79.70) 271 (20.30)
Pain duration	
<1 year	42 (3.13)
1–4 years	303 (22.60)
5–9 years	302 (22.52)
≥10 years	694 (51.75)
Pain intensity (0–10 NRS) on average in the past 7 days	
Mild (1–4)	429 (32.30)
Moderate (5–7)	707 (53.24)
Severe (8–10)	192 (14.46)
Current use of prescribed pain medications Yes No	1073 (79.90) 270 (20.10)
Current use of over-the-counter pain medications Yes No	905 (67.39) 438 (32.61)
Most common locations of pain prevalence^b^	
Back	841 (62.57)
Neck	602 (44.79)
Shoulder	593 (44.12)
Hips	507 (37.72)
Legs	519 (38.62)
Multisite pain (two or more sites) Yes No	1192 (88.69) 152 (11.31)
Generalized pain Yes No	471 (35.04) 873 (64.96)

^a^Unless stated otherwise.

^b^Categories are not mutually exclusive.

NRS = numeric rating scale.

**Figure 1.**
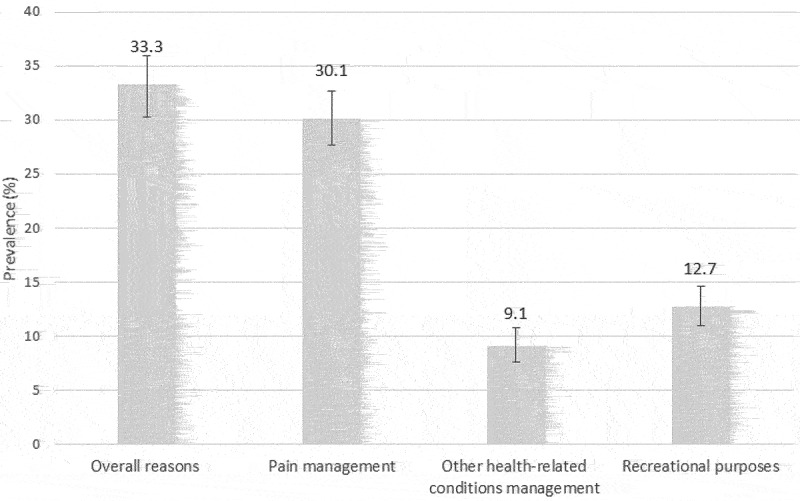
Prevalence of cannabis use according to reasons for use. Error bars represent 95% CIs. Non-mutually exclusive groups, because participants could check more than one reason.

The overall prevalence of cannabis use among people living with CP was 33.3% (95% CI 30.7–35.8) (Figure 1). Prevalence of cannabis use included 30.1% (95% CI 27.7–32.7) of participants who reported using cannabis to manage pain, 9.1% (95% CI 7.6–10.8) to manage other health-related conditions, and 12.7% (95% CI 11.0–14.6) for recreational purposes (non-mutually exclusive groups, because participants could check more than one reason). In addition, 55.4% checked only one reason for cannabis use, 31.8% checked two reasons, and 12.8% checked all three reasons (pain, other health-related conditions, and recreational purposes). Only 3.0% used cannabis for recreational purposes without selecting the pain management option. Although cannabis use was assessed using standardized questionnaire items, 56 participants also reported cannabis use when asked about nonpharmacologic treatments (in answer to a semi-open-ended question), allowing us to draw certain conclusions regarding how cannabis is represented among participants.

**Figure 2.**
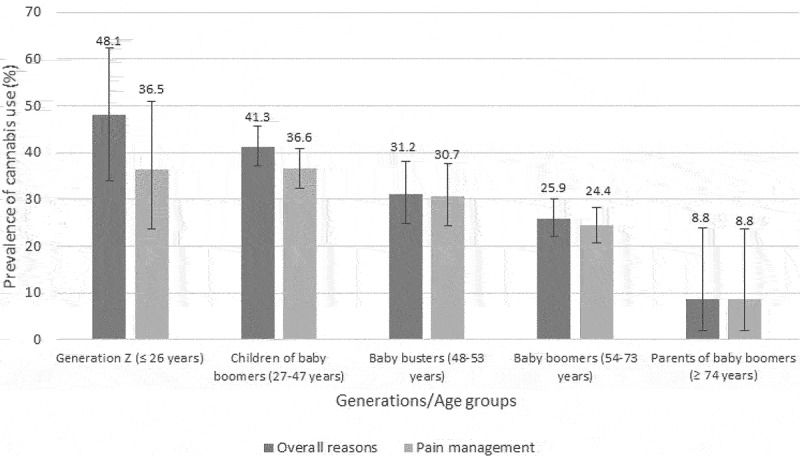
Prevalence of cannabis use per generations/age groups. Error bars represent 95% CIs.

**Figure 3.**
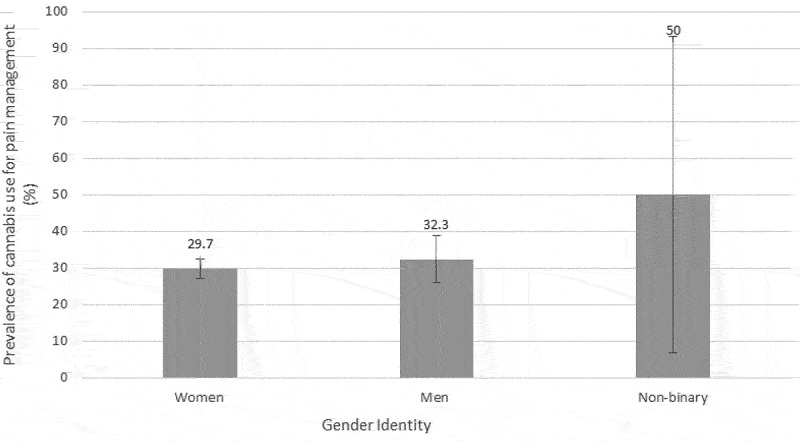
Prevalence of cannabis use for pain management according to gender identity. Error bars represent 95% CIs.

Figure 2 presents the prevalence of cannabis use overall and for pain management across generations. The proportion of participants reporting cannabis use for pain management significantly varied across age groups (*P* < 0.0001): 36.5% for Generation Z (≤26 years), 36.6% for children of baby boomers (27–47 years), 30.7% for baby busters (48–53 years), 24.4% for baby boomers (54–73 years), and 8.8% for the parents of baby boomers (≥74 years). Post hoc multiple comparisons revealed differences between (1) parents of baby boomers versus Generation Z, (2) parents of baby boomers versus children of baby boomers, (3) parents of baby boomers versus baby busters, and (4) baby boomers versus children of baby boomers. The prevalence of cannabis use for the management of pain was not found to significantly vary across gender identity groups (*P* = 0.4253; see Figure 3).

Table 2 shows the estimates of the multivariable model used to investigate the relationship between age and cannabis use for CP management. Independent of other sociodemographic and clinical factors included in the model, being older was associated with a decreased likelihood of using cannabis to manage CP symptoms; Compared to Generation Z, baby busters (OR = 0.4, 95% CI 0.2–0.9), baby boomers (OR = 0.3, 95% CI 0.1–0.6), and parents of baby boomers (OR = 0.1, 95% CI 0.01–0.8) were less likely to use cannabis for CP management. Neither missing data imputation nor entering age in the model as a continuous versus categorical variable changed that conclusion.

**Table 2.** Results from the multivariate analysis with adjusted ORs and their 95% CIs.

	OR	95% CI	
*Sociodemographic profile*			
Generation/age group (versus Generation Z)			
Children of baby boomers (27–47 years)	0.622	0.307	1.260
Baby busters (48–53 years)	**0.405**	**0.185**	**0.886**
Baby boomers (54–73 years)	**0.271**	**0.126**	**0.581**
Parents of baby boomers (≥74 years)	**0.086**	**0.010**	**0.780**
Self-identified gender (women versus other)^a^	0.812	0.533	1.236
Gender role according to the BSRI (versus undifferentiated)			
Feminine	1.240	0.810	1.898
Masculine	1.217	0.788	1.882
Androgynous	1.090	0.728	1.635
Country of birth (Canada versus other)	1.175	0.512	2.695
Aboriginal (yes versus no)	1.208	0.374	3.903
Employed full- or part-time (yes versus no)	0.821	0.580	1.162
Disabled (yes versus no)	0.889	0.579	1.365
Postsecondary education (yes versus no)	1.406	0.938	2.107
Residing in a remote region (versus nonremote regions)^b^	**0.636**	**0.436**	**0.928**
*Chronic pain characteristics and interference*			
Generalized pain (yes versus no)	**1.604**	**1.148**	**2.242**
Multisite pain (yes versus no)	1.251	0.714	2.194
Frequency (continuous versus intermittent)	1.029	0.610	1.735
Duration (years)	1.003	0.989	1.017
Pain intensity on average in the past 7 days (0–10 NRS)	0.926	0.838	1.025
Tendency to pain catastrophizing (yes versus no)	1.189	0.836	1.693
Neuropathic component according to the DN4 (yes versus no)	1.297	0.940	1.787
Interference (BPI score)	1.081	0.967	1.209
*Pain treatment*			
Using prescribed pain medications (yes versus no)	**1.783**	**1.127**	**2.820**
Using over-the-counter pain medications (yes versus no)	**0.639**	**0.469**	**0.870**
Using nonpharmacological treatments for pain management (yes versus no)	**1.628**	**1.053**	**2.517**
Access to a trusted health care professional for pain management (yes versus no)	1.256	0.854	1.847
*Health profile and lifestyle*			
Psychological distress (0–12 PHQ-4 score; versus none/scores 0–2)			
Mild/scores 3–5	1.208	0.789	1.850
Moderate/scores 6–8	1.036	0.638	1.683
Severe/scores 9–12	0.849	0.488	1.476
Perceived general health (0–100 SF-12 score)	0.991	0.977	1.005
Number of medications currently used (including prescribed, over-the-counter, pain-related, and non-pain-related)	0.998	0.960	1.037
Physical functioning (0–100 SF-12 score)	1.000	0.981	1.019
Have consumed alcohol or used drugs more than intended in the past year (versus never)			
Rarely	**2.047**	**1.434**	**2.923**
Sometimes	**3.047**	**1.967**	**4.719**
Often	**3.017**	**1.595**	**5.706**
Cigarette smoking (versus never smoked)			
Current smoker	**3.601**	**2.315**	**5.601**
Former smoker	**1.870**	**1.339**	**2.611**

Proportion of missing data across presented variable ranges between 0.07% and 8.85%. Listwise deletion led to the inclusion of 1068 out of 1344 individuals in the final model. Bold font indicates statistical significance.

^a^Men and participants who self-identified as unknown or unspecified sex were regrouped because the latter included only four individuals.

^b^Remote resource regions as defined by Revenu Quebec (i.e., the provincial revenue agency): Bas-Saint-Laurent, Saguenay–Lac-Saint-Jean, Abitibi-Témiscamingue, Côte-Nord, Nord-du-Québec, Gaspésie–Îles-de-la-Madeleine. Nonremote regions are near a major urban center.

BPI = Brief Pain Inventory; DN4 = Douleur Neuropathique 4; NRS = numeric rating scale; PHQ-4 = 4-item Patient Health Questionnaire; SF-12 = 12-Item Short Form Survey.

Other factors associated with a decreased likelihood of using cannabis to manage CP symptoms were (1) residing in a remote region (OR = 0.6, 95% CI 0.4–0.9) and (2) using over-the-counter pain medications (OR = 0.6, 95% CI 0.5–0.9). Conversely, reporting generalized pain (OR = 1.6, 95% CI 1.2–2.2), using prescribed pain medications (OR = 1.8, 95% CI 1.1–2.8), using nonpharmacological treatments for pain management (OR = 1.6, 95% CI 1.1–2.5), consuming alcohol or drugs more than intended (rarely versus never: OR = 2.0, 95% CI 1.4–2.9; sometimes versus never: OR = 3.0, 95% CI 2.0–4.7; often versus never: OR = 3.0, 95% CI 1.6–5.7), and being a current (OR = 3.6, 95% CI 2.3–5.6) or former (OR = 1.9, 95% CI 1.3–2.6) cigarette smoker (versus never smoked) were associated with an increased likelihood of using cannabis for pain management.

## Discussion

The present study took advantage of the large COPE cohort data set that includes more than one thousand adults from the province of Quebec (Canada) who are living with CP to estimate the post-legalization prevalence of cannabis use. The present results indicate an overall prevalence of cannabis use among Canadians living with CP of 33.3%, followed closely by a prevalence of 30.1% for pain management. Based on studies conducted before the legalization of recreational cannabis,^16,17^ the prevalence of cannabis use estimated in the present study indicates a threefold increase in reported usage. Cannabis use for CP management was especially frequent in younger generations, but no gender differences were found.

### Prevalence of Cannabis Use

Few cannabis use prevalence studies applied the same methodology among CP populations before and after legalization of recreational cannabis in Canada.^17,18^ In the general population, however, Statistics Canada reported an increase in cannabis use from approximatively 15% to 17% between 2018 and 2019.^44^ It was also reported in the Canadian Cannabis Survey 2020 that 27% of Canadians had used cannabis in the past 12 months (compared to 22% in 2018).^5^ An international study showed that frequency of adults reporting having ever used cannabis for medical purposes in the United States in 2018 was 34% in states where recrational use is legal, 23% in states where it is illegal, 25% in states where medical use only is legal; in Canada, the frequency was 25%.^45^ In a study published post-legalization and conducted among people living with fibromyalgia, Fitzcharles and colleagues^46^ found a prevalence of cannabis use of 23.9%. The prevalence of cannabis use was higher in our study (33.3% in general and 30.1% for pain management in 2019). Because people living with CP have reported using even more cannabis during the first wave of the COVID-19 pandemic,^47^ it seems reasonable to expect the prevalence to be even higher today. Our results suggest that cannabis is a common treatment reported by people living with CP and underscore the importance of rapidly generating more evidence on the safety and efficacy of cannabis. In addition, some participants described cannabis use when asked about nonpharmacologic treatments, which suggests that the pharmaceutical properties of cannabis are underestimated. In fact, cannabis is often considered natural and safe and not a drug.^48,49^ In essence, there is an urgent need for better knowledge translation for people living with CP and engagement of community-based clinicians in supporting people who use medical as well as recreational cannabis for pain management. Above all, generating high-quality evidence on the safety and efficacy of cannabis use among people living with CP is a priority.

Legalization of recreational cannabis in Canada has created a situation where it can be used for treatment without having gone through rigorous drug approval process. Security is a challenge because patients can self-medicate without proper advice from a health care professional, employees of government-operated recreational cannabis are not authorized to give advice on medicinal use, and cannabis use is not systematically recorded in medical and pharmacy charts. Even in regions where cannabis is legal, people do not always disclose cannabis use to health care professionals for a variety of reasons, including a perceived stigma, unfavorable attitudes of health care professionals, not wanting cannabis in their medical chart, fear of losing professional status, and fear of losing health insurance.^50^ This self-medication raises major concerns regarding drug–drug interactions and management of adverse effects. In terms of support, it has been argued that pharmacists should be providing counsel to medicinal cannabis users^51,52^ and potentially should be the dispensers of medical cannabis in Canada.^53^ A recent survey of individuals living with CP suggested that more than half would prefer their treating physician to be involved in the prescription of cannabis products.^13^ More studies about the expected versus actual versus desired role of primary care health care professionals should be conducted. Given the most recent position statements from the International Association for the Study of Pain Presidential Task Force^8^ and the Canadian Rheumatology Association,^10^ which do not currently support the use of cannabis and cannabinoids for the purpose of pain management, it is not likely that people will disclose their use of cannabis to their health care professionals. This lack of communication can lead to unintended consequences. For instance, cannabis use in people living with CP who are prescribed a chronic opioid therapy has been associated with opioid misuse.^54^ Therefore, if professionals were better informed of patients’ use of cannabis, they could work with patients appropriately by providing sound advice regarding pain management. It is thus essential to rapidly put in place awareness and education campaigns for patients and health care professionals and to encourage professionals to have an open and honest discussion with their patients regarding their use of cannabis products.

### Reasons of Consumption

Though 30.1% of our participants with CP used cannabis for pain management and 12.7% reported using it for recreational purposes, these categories were not mutually exclusive, and only few participants used cannabis for recreational purposes without pain management goals (3%). To our knowledge, no other study specifically evaluated recreational cannabis use post-legalization in Canadians living with CP. Our results further emphasize the importance of better support for people living with CP. Because the reasons for use and the rationale for self-medication may vary compared to the general population or people using cannabis for other medical reasons, we suggest that efforts be tailored to the CP population. Specific “other health-related conditions” were not listed by participants in our study, but results of recent Canadian studies among medical and recreational cannabis users suggest that, in addition to pain, anxiety, insomnia, and depression are common conditions for which cannabis is used.^3,55^

### Differences across Age Groups

One aim of the present study was to explore whether there were differences in cannabis use for CP management between age groups. In fact, new legislation and age, alongside sex, ethnicity, and socioeconomic status, can have an influence on cannabis use trends^19^ and are important to take into consideration to understand the real context of cannabis use in Canada. In our study, a higher prevalence of cannabis use for CP management was observed among younger generations, whereas people aged 74 years or older (parents of baby boomers) reported the lowest use. Baby boomers did not stand out in terms of cannabis use, although cannabis use was more prevalent among this generation in the 1960s and 1970s, which could be a factor influencing openness toward cannabis use.^56^ Our results are in line with other studies suggesting that cannabis use is higher in younger age groups and not often use in those aged 75 years and older (both in CP populations^10,57^ and in the general Canadian population^58^). The age groups with the highest cannabis use were those ≤26 and 27–47 years old, in accordance with previous investigations. This higher prevalence of cannabis use among youth could be partly explained by the fact that younger generations often have fewer responsibilities (cannabis use decreases with additional responsibilities such as work and family^59^) or because of peer influence.^59,60^ It could also be related to vaping habits among youth.^61^ Because cannabis use at a younger age has been associated with multiple subsequent adverse health and social effects,^62^ it is concerning to see such a high prevalence of cannabis use among the younger generations in Canada. Globally, knowledge translation implementation and messages for people living with CP should be tailored according to age.

### Absence of Gender Differences

Some studies of people living with CP showed a lower prevalence of cannabis use among women.^57^ Others found that women were more likely to substitute cannabis for analgesics.^63^ Differences have also been noted in the general Canadian population. In fact, in 2013 the prevalence of cannabis use was nearly double among men compared to women (13.9% versus 7.4%).^64^ Similar differences between men and women were found in 2020 (31% versus 23%).^5^ In contrast to these reports, our study revealed that prevalence of cannabis use for pain management was not statistically different between men (32.3%) and women (29.7%). Multivariable analyses supported the absence of gender identity and gender role differences. Comprehensive sex- and gender-based analyses^65–67^ of cannabis use among people living with CP could complete our findings.

### Other Factors Associated with Cannabis Use for Pain Management

It is beyond the scope of this article to discuss all of the factors associated with cannabis use for pain management. However, some of these factors (Table 2) are worth mentioning and should be addressed by future studies: residing in a remote region (associated with decreased use) and using nonpharmacological treatments for pain management (associated with increased use).

### Strengths and Limitations

The present study has a number of important strengths, such as the use of a large community sample of adults living with CP, which enhances statistical power of the analyses, and the inclusion of a broad set of variables relevant to the CP experience. Some limitations must nonetheless be highlighted. First, participants were self-selected and all data were self-reported. Although cannabis use has become more socially acceptable, it is possible that some participants did not disclose their use due to social desirability concerns. This may be especially true in older age groups. It is thus possible that prevalence of cannabis use among persons living with CP is even higher that that estimated in our study. However, this limitation is not unique to the present prevalence study and thus does not undermine its ability to suggest an increase in self-reported prevalence of cannabis use for recreational and/or pain management reasons. Second, the COPE database was not specifically designed for cannabis research. With the available data, it was thus not possible to differentiate between medical cannabis and recreational cannabis users or type of product, mode of administration, and directions for use. Such differentiation would have been interesting, and we acknowledge that prevalence of use could vary according to such cannabis product characteristics. However, this does not affect the relevance of our recommendations, which are applicable to cannabis regardless of the channel through which it is obtained. Third, we found no gender identity differences in the proportion of cannabis users, suggesting valid prevalence estimates. Finally, data were cross-sectional, which limits our ability to make causal assumptions, detect changes in cannabis use in this specific population, or identify predictors of increased/decreased use. Future longitudinal research would be important to study trajectories of cannabis use over time.

### Conclusions

The present study suggests a higher prevalence of cannabis use for pain management among people living with CP since its legalization for recreational purposes in 2018. Our results also indicate a higher prevalence of cannabis use for pain management among younger generations, whereas people aged 74 years or older reported significantly lower use. Cannabis is thus a common treatment reported in people living with CP, and its legalization in Canada has created a situation where it can be used for self-medication without having gone through rigorous drug approval processes and without proper guidance from patients’ health care teams. Our study re-emphasizes the importance of rapidly generating evidence on the safety and effectiveness of cannabis, in addition to age-tailored education and awareness efforts among people living with CP.

## Data Availability

The data supporting the findings of this study are available from the corresponding author, A. Lacasse, upon reasonable request and conditional to proper ethics approval for a secondary data analysis. The data are not publicly available because participants did not provide consent to open data.
